# Balancing the immune response in the brain: IL-10 and its regulation

**DOI:** 10.1186/s12974-016-0763-8

**Published:** 2016-11-24

**Authors:** Diogo Lobo-Silva, Guilhermina M. Carriche, A. Gil Castro, Susana Roque, Margarida Saraiva

**Affiliations:** 1 Life and Health Sciences Research Institute (ICVS), School of Health Sciences, University of Minho, Braga, Portugal; 2 ICVS/3B’s PT Government Associate Laboratory, Braga, Portugal; 3 i3S - Instituto de Investigação e Inovação em Saúde, Universidade do Porto, Rua Alfredo Allen, 208, 4200-135 Porto, Portugal; 4 IBMC – Instituto de Biologia Molecular e Celular, Universidade do Porto, Porto, Portugal

**Keywords:** Interleukin-10, Pattern recognition receptors, Glial cells, Molecular regulation, Neurodegeneration

## Abstract

**Background:**

The inflammatory response is critical to fight insults, such as pathogen invasion or tissue damage, but if not resolved often becomes detrimental to the host. A growing body of evidence places non-resolved inflammation at the core of various pathologies, from cancer to neurodegenerative diseases. It is therefore not surprising that the immune system has evolved several regulatory mechanisms to achieve maximum protection in the absence of pathology.

**Main body:**

The production of the anti-inflammatory cytokine interleukin (IL)-10 is one of the most important mechanisms evolved by many immune cells to counteract damage driven by excessive inflammation. Innate immune cells of the central nervous system, notably microglia, are no exception and produce IL-10 downstream of pattern recognition receptors activation. However, whereas the molecular mechanisms regulating IL-10 expression by innate and acquired immune cells of the periphery have been extensively addressed, our knowledge on the modulation of IL-10 expression by central nervous cells is much scattered. This review addresses the current understanding on the molecular mechanisms regulating IL-10 expression by innate immune cells of the brain and the implications of IL-10 modulation in neurodegenerative disorders.

**Conclusion:**

The regulation of IL-10 production by central nervous cells remains a challenging field. Answering the many remaining outstanding questions will contribute to the design of targeted approaches aiming at controlling deleterious inflammation in the brain.

## Background

The process of microglial cells and astrocyte activation is an essential component of the inflammatory response against pathogens and damage in the central nervous system (CNS). However, if not regulated, this process leads to a series of events that culminate in the damage of healthy nearby cells. In this context, the tight regulation of immune cell activation is of utmost importance, to avoid propagation of neurodegenerative processes. Interleukin (IL)-10, an anti-inflammatory cytokine, prevents immunopathology in several diseases and disease models, both in the periphery and in the CNS. Here, we review the current knowledge on the molecular mechanisms regulating IL-10 production by microglial cells and astrocytes and how that balances the immune response in the CNS.

## Introduction

Interleukin (IL)-10 was identified over two decades ago [1] and is to date the most studied suppressive molecule of the immune system. IL-10 plays a critical role in preventing inflammatory and autoimmune pathologies by limiting the immune response to pathogens and microbial flora [2]. Mouse models of IL-10 deficiency develop inflammatory bowel disease upon colonization of the gut with particular microorganisms [3], whereas in humans, genetic studies have confirmed the essential role of IL-10 in preventing deleterious inflammation in the gut [4]. However, the role of IL-10 clearly exceeds the regulation of intestinal inflammation, as a function for this molecule has been also described in chronic infection, tumour surveillance and neurodegenerative disorders [5–7].

IL-10 production was originally ascribed to CD4+ T helper type 2 (Th2) cells [1]. Since then, IL-10 production has been described for a wide variety of immune cells, including Th1 and regulatory T cells, CD8+ T cells, B cells, macrophages, dendritic cells, neutrophils and eosinophils [6]. Notably, some non-haematopoietic cells, as epithelial cells, are also able to produce IL-10 [8]. The modulation of IL-10 expression by different cells is complex, with common and cell-specific regulatory molecular mechanisms in place [6]. These mechanisms include epigenetic regulation, the expression and activation of particular transcription factors, the triggering of signalling pathways, and post-transcriptional regulation [6, 9]. It is now evident that deregulation of these fine-tuned processes is associated with detrimental effects of IL-10. As such, understanding the regulation of IL-10 expression by different cells is instrumental for the targeted design of immune intervention strategies.

IL-10 production in the brain has also been described, but the cellular sources and regulatory molecular mechanisms involved are much less known than those operating at the periphery. Considering the potential of IL-10 in regulating the immune response in the brain, this lack of knowledge hampers the development of novel immune modulatory strategies. In this review, we start by providing a global view on the immune response in the central nervous system (CNS) and of the potential interest of IL-10 regulation in the context of neuroinflammation versus neurodegeneration and then discuss our current understanding on the molecular mechanisms underlying IL-10 production by CNS cells.

## Global concepts on the immune response in the CNS

Although for decades the CNS has been considered an immune privileged site due to the believed absence of a local immune response, extensive work in the last decade unravelled the presence of a specialized intrinsic innate immune system in the CNS [10, 11]. Indeed, we now know that immune surveillance actively occurs in the CNS and that its well-functioning is fundamental to maintain the CNS homeostasis [10, 11]. Furthermore, classical lymphatic vessels were recently discovered in the CNS *dura mater* [12, 13], establishing a direct interface to the peripheral immune system. Whereas the resident innate immune system patrols the CNS as a first line of defence, the presence of the adaptive immune response is controlled by a series of interfaces that include the brain-blood-barrier and the choroid plexus that likely represent a protective measure against immune-mediated damage [14].

The innate immune response is generally classified as the first line of defence against pathogens, being fast and relatively non-specific [15, 16]. Innate immune cells express a wide variety of pattern recognition receptors (PRRs) which, upon recognition of danger signals, trigger a series of intracellular cascades that culminate with the production of immune mediators, among which pro- and anti-inflammatory cytokines [15]. Collectively, PRRs recognize pathogen-associated molecular patterns (PAMPs) present in pathogens and damage-associated molecular patterns (DAMPs) released by tissue damage in the absence of infection [16]. Whereas recognition of PAMPs initiates an immune response aimed at pathogen clearance, activation of PRRs via DAMPs aims at the resolution of tissue damage [15]. Recognition of PAMPs and DAMPs are critical for the organism homeostasis, and in both cases, the regulation of the initial immune response is needed, to avoid collateral tissue damage. Several cells of the CNS, including microglia, astrocytes, neurons, neural stem cells and endothelial cells express PRRs [11, 17–20], therefore contributing to the initiation of the innate immune response. The best-studied cellular population in this context is by far microglia.

Microglia are the resident macrophages of the brain and the only CNS cells of haematopoietic origin [21]. Microglia arise from the yolk sac-derived primitive macrophage population and migrate to the brain early in development, completing their maturation in the forming CNS [22, 23]. They are thus distinct from other glial cells (i.e. astrocytes and oligodendrocytes) and neurons, which are derived from the neuroectoderm [24], and a unique population among mononuclear phagocytes. Microglia are capable of self-renewal and do not appear to require replenishment from circulating bone marrow-originated monocytic precursors [25]. Nevertheless, in inflammatory conditions such as in experimental autoimmune encephalitis (EAE), circulating monocytes are recruited to the CNS, where they remain functionally distinct from microglia, and participate in disease progression [26, 27]. These infiltrating monocytes only contribute to the CNS myeloid cell pool in a transient manner [27]. In line with their myeloid origin, microglia express an array of PRRs and signalling molecules that allow their response to perturbations in brain homeostasis, namely with the production of several cytokines, chemokines and reactive oxygen and nitrogen species [28, 29]. This response initially provides the environment needed for neuronal regeneration and functional recovery, thus being protective in its nature. However, a deregulated or non-resolving microglial inflammatory response may lead to the uncontrolled production of immune mediators and recruitment of peripheral immune cell populations, which induce secondary damage to intact tissue and inhibit post-injury CNS repair [27, 30]. Indeed, deregulated microglial responses have been increasingly associated with a series of neurodegenerative disorders, from Alzheimer’s and Parkinson’s diseases to multiple sclerosis [29, 31, 32]. The classical view of microglia as the sole innate immune cells of the CNS has been challenged by findings that place astrocytes as immune players, with a critical role in the formation of the glial scar and tissue integrity restoration [33]. Similarly to microglial cells, astrocytes express PRRs, namely surface-expressed toll-like receptors (TLRs), which upon activation lead to the production of several inflammatory mediators [34, 35]. Finally, neurons, oligodendrocytes and endothelial cells also express PRRs [35–38] and therefore have the capacity to contribute to the inflammatory response in the brain.

## Neuroprotection vs neurodegeneration: a role for IL-10 in tipping this balance

**Fig. 1:**
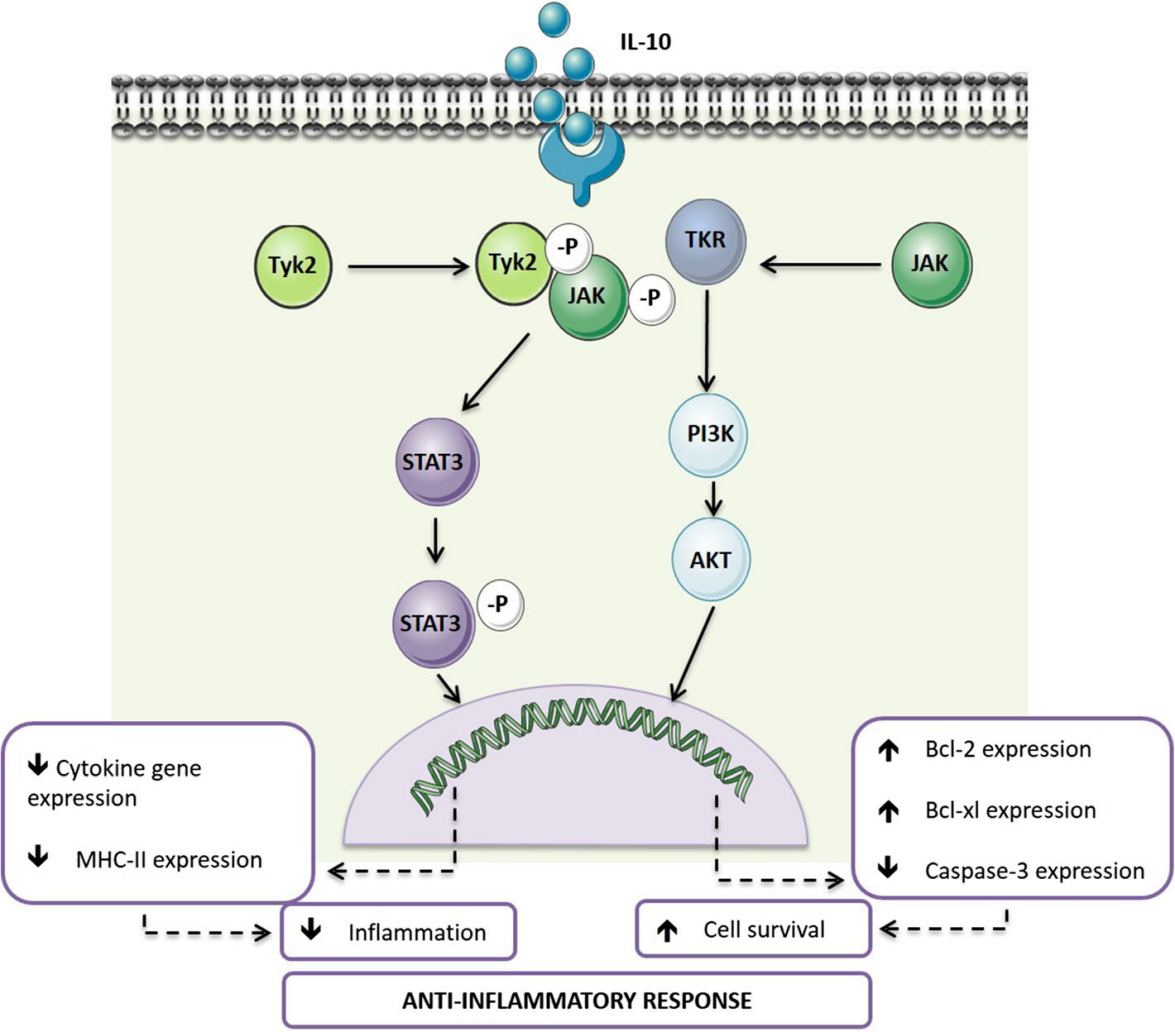
The role of IL-10 receptor signalling in anti-inflammation. Overview of the IL-10R signalling cascade and the main cellular effects triggered by IL-10

The manipulation of the balance between protective and degenerative neuroinflammation is gaining importance from a therapeutic point of view. Among the mechanisms in place to avoid exaggerated neuroimmune responses is the production of anti-inflammatory cytokines, such as IL-10. Binding of IL-10 to its receptor triggers a series of signalling cascades mediated by the Janus kinase (JAK) signal transducer and activator of transcription (STAT) pathway, particularly by STAT3 [39]. Signalling through the IL-10 receptor regulates several steps of the immune response, from decreasing cytokine gene expression to down-regulating the expression of major histocompatibility complex class II (MHC-II) and thus antigen presentation to T cells [39, 40]. Furthermore, IL-10 has been shown to prevent apoptosis by activating the phosphatidylinositol-4,5-bisphosphate-3-kinase (PI3K)/Akt cascade and enhancing the expression of anti-apoptotic factors as Bcl-2 and Bcl-xl, whilst attenuating that of caspase-3 [40]. The processes mediated by IL-10 (Fig. 1) have important implications at the CNS level. IL-10 is able to inhibit the production of pro-inflammatory cytokines by microglia, protecting astrocytes from excessive inflammation [41, 42]. IL-10 also acts on astrocytes by potentiating their production of transforming growth factor (TGF)-β [43]. In neurons, IL-10 receptor signalling has been associated with increased cellular survival [44, 45] and the regulation of adult neurogenesis [46, 47]. Thus, IL-10 is an important mediator of the crosstalk between microglia, astrocytes and neurons. Importantly, in addition to the evidence placing IL-10 as a regulator of the immune crosstalk in the CNS, several studies directly implicate defective IL-10 production or signalling in patients and animal models of neurological diseases, ranging from neuropathic pain [48] to multiple sclerosis [49, 50], Alzheimer’s disease [51] or Parkinson’s disease [52].

Microglia continuously survey the surrounding tissue environment, responding to any disturbance in neuronal homeostasis [53–55]. Depending on the detected insult and on the alterations to the microenvironmental niche, a spectrum of activated microglia results. The two extremes of this spectrum are M1 and M2 polarized cells, as described for macrophages of the periphery [56]. Upon detection of tissue injury or infection, or upon activation of microglial cultures with lipopolysaccharide (LPS) and interferon (IFN)-γ, necrotic neurons, oligomers of amyloid-β and α-synuclein, microglia acquire an activated phenotype, typically referred to as classical or M1. This phenotype is characterized by the production of pro-inflammatory mediators, including cytokines, such as tumour necrosis factor (TNF), IL-6 and IL-1β; chemokines; reactive oxygen species; nitric oxide; and prostaglandins [57]. Although important to fight infections or injury, most of the factors released by M1 microglia are toxic for neuronal cell cultures. At the other end of the spectrum, in the presence of IL-4, IL-13 or IL-10, microglia differentiate into M2 or alternative phenotype, characterized by the expression of IL-10; heparin-binding lectin (Ym1); cysteine-rich protein FIZZ-1; and Arginase 1 [57]. The anti-inflammatory M2 phenotype is neuroprotective and has been implicated in the resolution of the inflammation, phagocytosis and tissue repair. In recent years, the M1/M2 paradigm of microglial activation has been increasingly studied in several neurodegenerative and neurological diseases, where an imbalance towards M1 polarization is documented [58, 59]. Therefore, normalizing the imbalance between M1 and M2 microglial polarization states has been proposed as a therapeutic target for the treatment of several CNS-linked disorders [60, 61]. In this context, in cell cultures, IL-10 in combination with IL-13 increased microglial secretion of activin-A, a neuroprotective TGF-β superfamily member that promotes oligodendrocyte differentiation [62]. More recently, in vivo, IL-10 originated from regulatory T cells was shown to mediate microglia polarization towards the M2 phenotype ameliorating the outcome of intracerebral haemorrhage [63]. Other approaches to drive M2 microglia include the administration of anti-inflammatory agents [64], which action may well be mediated by IL-10, as they are known to enhance IL-10 expression, as discussed below.

Taking all this together, a variety of methods to increase IL-10 and test its therapeutic potential in neurological disorders with an immune component have been developed. These include the administration of recombinant IL-10, the enhancement of IL-10 production through agonists, the delivery of IL-10 through viral vectors or the potentiation of IL-10-producing T and B regulatory cells [7, 65, 66]. However, despite the initially high expectations, the therapeutic success of IL-10 has been conflicting. Taking the case of multiple sclerosis as an example, the effect of IL-10 administration during EAE, varies from decreased, to no effect, to actual increased clinical scores [7, 67]. Several factors may explain the variability of outcomes upon IL-10-targeted interventions in EAE. Different studies use different routes of IL-10 administration, thus possibly compromising the bioavailability of IL-10 in the needed anatomical location. This is illustrated by the fact that intracranial administration of IL-10 improved the outcome of EAE [68], whereas systemic delivery did not [69]. Also, IL-10 appears to be generally more effective if delivered as gene therapy directly into the CNS than as recombinant protein [7], which again may reflect the bioavailability of the molecule. Finally, data also suggest that IL-10 enhancement at disease onset, rather than later on, produced the best effects [66]. It is conceivable that with disease progression both IL-10-producing and IL-10-responding cells change, and so the action of IL-10 also varies with time. In a recent study, nasal administration of a CD3-specific antibody was shown to ameliorate EAE in an IL-10-dependent manner [65], supporting the concept that IL-10 activating therapies may also be of interest. The observations reported in the context of EAE find parallel in other diseases where IL-10 appears as a possible therapeutic target, as recently reviewed by Kwilasz et al. [7]. To mention a few examples, a role for IL-10 in attenuating local inflammatory reactions during permanent ischemia has been described [70]. Also, adenoviral-mediated expression of human IL-10 in the striatum of mice subjected to MPTP (1-methyl-4-phenyl-1,2,3,6-tetrahydropyridine) neurotoxicant model of Parkinson’s disease decreased the neurodegenerative effect of MPTP administration [71]. Importantly, the benefits of dampening neuroinflammation are highly dependent on the disease type. In the context of Alzheimer’s disease, several studies show that skewing innate immunity towards a pro-inflammatory state reduce amyloid-β deposition in transgenic mouse models, via enhancing amyloid-β clearance by microglia [72–78]. In line with these findings, administration of IL-10 to the brains of amyloid precursor protein (APP) transgenic mice led to amyloid-β accumulation accompanied by reduced amyloid-β phagocytosis by microglia [79]. The detrimental effects of IL-10 in Alzheimer’s disease were further confirmed as IL-10 absence in a transgenic mouse model of cerebral amyloidosis resulted in amyloid-β phagocytosis by activated microglia and in reduced amyloid-β load in the mouse brains, which ultimately conferred a better outcome of the disease [80]. Therefore, rebalancing the cerebral innate immunity by inhibiting actions of key anti-inflammatory cytokines, such as IL-10, to allow the brain return to a physiological state may also be a potential therapeutic strategy [80]. Thus, rewiring the immune response, at the functional, but also temporal and spatial levels, more than simply blocking inflammation, may prove a better approach to resolve the neuroimmune element of neurodegeneration. Therefore, a deep understanding of the cellular and molecular mechanisms operating to regulate the production of IL-10 is critical.

## Cellular sources of IL-10 in the CNS

Microglial cells are the most investigated innate immune cells in the brain and thus the main studied cytokine producers, including of IL-10. Studies performed in vitro show that IL-10 production is induced in microglial cells upon TLR stimulation. Specifically, microglial cells produced IL-10 upon TLR2, 3, 4 and 9 stimulation [34, 41, 81–84]. TLR-induced IL-10 production by microglia can be further regulated by other molecules. This is the case of adenosine that enhances the production of IL-10 by microglial cells upon stimulation of TLR2 and 4, whilst down-regulating the production of pro-inflammatory cytokines [85]. In vitro activation of purinergic receptors by extracellular ATP, either LPS-induced or exogenously provided, induced the production of IL-10 [86, 87]. Glutamate, the most common excitatory neurotransmitter, has also been shown to enhance the expression of IL-10 by LPS-stimulated microglial cells [81]. Prostaglandin E_2_, an immune mediator present in inflammatory settings, is another example of molecules that enhance LPS-induced IL-10 expression and secretion [88]. Benfotiamine, a synthetic vitamin B1 derivate, downregulates the production of pro-inflammatory cytokines by TLR4-stimulated BV2 microglia cell line, whereas up-regulating that of IL-10 [89]. Mycoepoxydiene was also shown to potentiate IL-10 production by TLR4-activated microglial cells, therefore reducing inflammatory markers [90]. Endocannabinoids enhance IL-10 production by activated microglia [91], as some anti-inflammatory drugs do [92]. Cytokines have also been described to impact IL-10 production by microglia. For example, tumour necrosis factor receptor 2 (TNFR2) triggering upon TLR4-induced TNF was shown to upregulate IL-10 production, which in this context was inhibited if in the presence of IFN-γ [93]. This last observation has, however, been contradicted in another study showing that Th1 cell-derived IFN-γ upregulates IL-10 production by microglial cells [94]. The production of IL-10 by microglia can also be induced in a PRR-independent manner. Glatiramer acetate, an immunomodulatory agent used in relapsing-remitting multiple sclerosis, was shown to induce secretion of IL-10 by microglial cells [95]. Resveratrol administration to a microglial cell line prevented hypoxia-induced injury by up-regulating IL-10 and controlling inflammation [96]. Collectively, these reports show that the molecules present in the microenvironment can impact IL-10 production by microglia, thus shaping the outcome of the inflammatory response. In addition to microglia, other CNS cells were shown to produce IL-10. Astrocytes produce IL-10 in response to PAMPs, as demonstrated in the case of the synthetic TLR3 ligand (double-stranded RNA) [97], *Neisseria meningitidis* or *Borrelia burgdorferi* [98], HIV membrane proteins [99] or human herpesvirus 6 if in the presence of IL-1β, TNF or IFN-γ [100]. Notably, stimulation of the Fcγ receptor I on the surface of astrocytes also triggers an immune response, with production of IL-10 [101].

Identification of cellular sources of IL-10 in vivo is more difficult than in vitro, but many studies report the presence of IL-10 in the CNS. In vivo TLR4 stimulation upon peripheral or central injection of LPS into wild-type mice, leads to IL-10 production [52, 102, 103]. IL-10 was also shown to be elevated in the injured adult brain in several neurodegenerative diseases and animal models of disease, such as excitotoxic shock [104], multiple sclerosis [105, 106], EAE [107–109], middle cerebral artery occlusion [110], traumatic brain injury [111], Alzheimer’s disease [112] and Parkinson’s disease [113]. Although much is yet to know concerning IL-10 induction in the injured brain, in some situations it involved TLR signalling, supporting the importance of TLRs in inducing IL-10 in the brain. For example, IL-10 production during Alzheimer’s disease occurs through a mechanism involving TLR4 [114]. In line with in vitro studies, anti-inflammatory drugs induce IL-10 production in vivo. In an experimental model of intracerebral haemorrhage, atorvastatin, a 3-hydroxy-3-methyl-glutaryl-coenzyme A reductase inhibitor with known anti-inflammatory properties, upregulated IL-10 in vivo, which is accompanied by a reduction in the number of activated microglial cells, and consequently to downregulate that of TNF [115].

Finally, it is important to refer that much of the immune mediators, including IL-10, present within the CNS, in the context of disease, are produced by brain-infiltrating immune cells. Initial activation of microglia results in the production of several pro-inflammatory mediators, which favour the brain-blood-barrier permeabilization and, ultimately, drive the infiltration of peripheral leukocytes into the CNS, including macrophages and T cells. Since macrophages, as microglia, detect PAMPs and DAMPs through the activation of PPRs, their arrival to the CNS will contribute to the full immune response established, either through the direct production of cytokines or indirectly through the modulation of the tissue microenvironment faced by microglia [116]. Likewise, infiltrating T cells may alter the cytokine *milieu* and thus reprogram microglia responses into M1 or M2 phenotypes [116]. Therefore, peripheral immune cells play a relevant role in the outcome of neuroinflammation associated with neurologic disorders.

## Molecular mechanisms regulating IL-10 production in the CNS

Gene expression requires the accessibility of transcription factors to DNA, which is achieved through chromatin remodelling events [117]. DNA methylation, nucleosome remodelling and covalent histone modifications, such as acetylation or methylation, are among the factors that influence the accessibility of chromatin [118]. Although the epigenetics of the *Il10* locus has been explored in macrophages, dendritic cells and T cells [6], to date it remains largely unknown in what concerns the regulation of IL-10 production by CNS cells. The level of methylation of the *Il10* gene was shown to be an important regulator of *Il10* mRNA transcription in microglial cells in the brains of rats subjected to morphine administration [119].

**Fig. 2:**
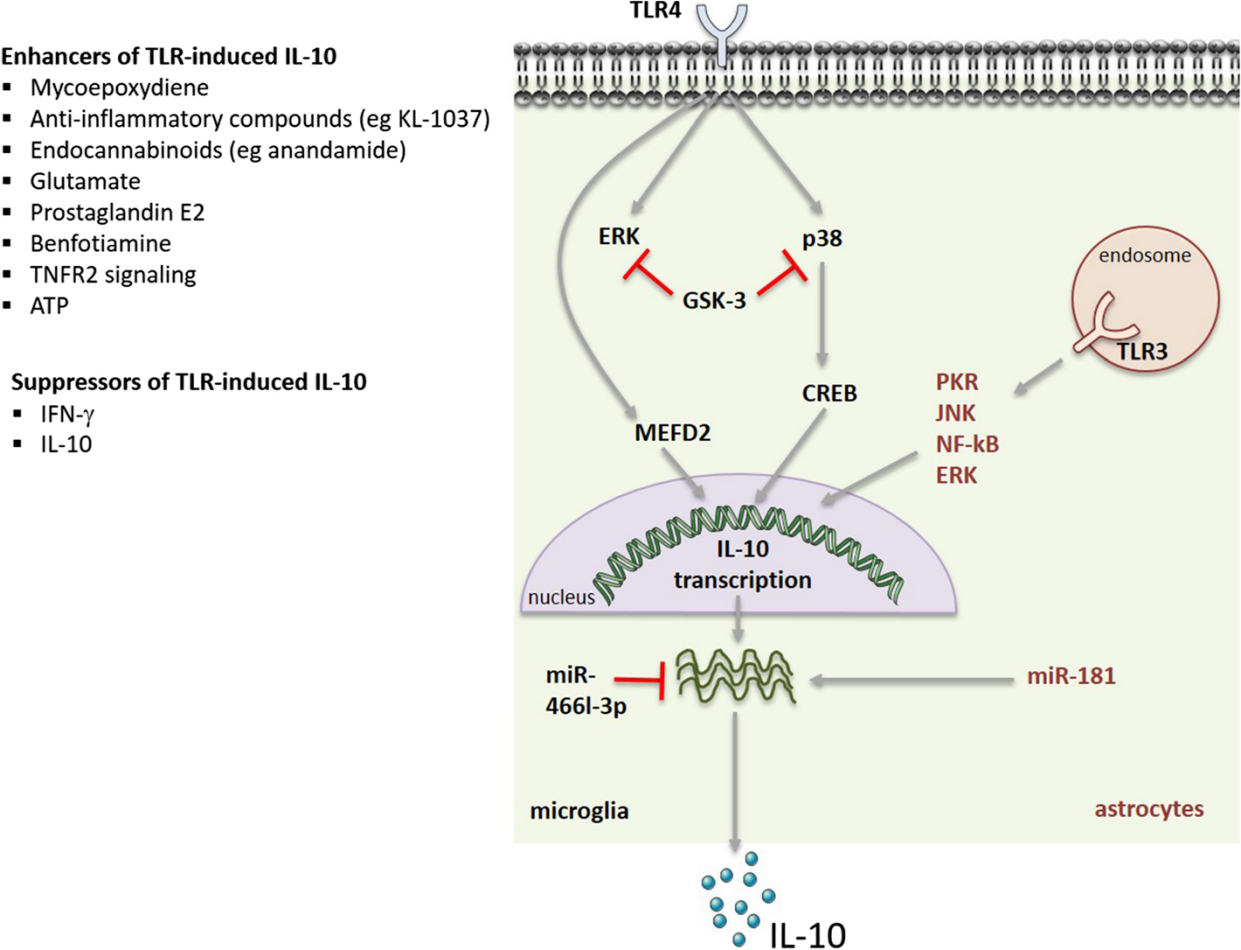
Molecular mechanisms regulating IL-10 production in microglia and astrocytes. Signalling cascades, transcription factors and miRNAs involved in regulating the production of IL-10 in TLR-triggered microglia or astrocytes. *Grey arrows* indicate positive signals; *red lines* indicate IL-10 silencing pathways. Also indicated are known enhancers and blockers of IL-10 production

Several intracellular signalling cascades are known to regulate the production of IL-10 by microglia and astrocytes (Fig. 2). Among them are the mitogen-activated protein kinase (MAPKs) extracellular signal-regulated kinase (ERK) and p38, which signal downstream of PRR activation and act as positive regulators of IL-10 production in myeloid cells [6]. ERK activation is also associated with the induction of IL-10 production by microglial cells upon TLR4 triggering and in the presence of some IL-10 enhancers [90–92, 120]. ERK activation was also reported to enhance IL-10 production by astrocytes upon TLR3 stimulation [97]. Conflicting reports exist in what concerns the role of p38 activation in IL-10 production by microglia. Activation of p38 has been shown to induce IL-10 expression in a microglial cell line upon adenosine administration [85]. However, in another study, a down-regulation on the activation of p38 in LPS-stimulated rat primary cultured microglia has been correlated with an enhancement in IL-10 prodution [121]. This controversy may be due to the different models used in both works, and/or to the distinct stimuli provided to microglial cells that may activate different molecular pathways. Another pathway identified as an inducer of IL-10 production by macrophages and dendritic cells is the tyrosine kinase Syk [6]. The upregulation of IL-10 upon in vivo adrenalin injection and transient opening of the blood-brain-barrier, associated with the upregulation of the FcγRI, involved the downstream activation of Syk [122]. Finally, the signalling molecules protein kinase R (PKR), c-Jun N-terminal kinase (JNK) and nuclear factor (NF)-κβ have been identified as likely players in regulating the Il-10 expression in TLR3-activated astrocytes [97]. Activation of IL-10 expression in astrocytes and neurons through HIV membrane proteins was shown to involve the PI3K pathway, through the activation of the serine/threonine kinase p70 [66, 99]. In all, several pathways have been involved in the regulation of IL-10 expression by microglia or astrocytes, illustrating the complexity of the system.

At the transcription factors level (Fig. 2), myocyte enhancer factor 2D (MEF2D) has been described to play an important role in activating IL-10 expression in microglia. MEF2D is present in microglial cells and increased upon microglia activation through TLR4, then binding to a MEF2 consensus site in the *Il10* promoter region, stimulating IL-10 transcription [123]. The transcription factor CREB was also shown to induce IL-10 expression downstream of p38 activation [85].

Post-transcriptional control of cytokine production is critical to ensure that the rapid transcription of cytokines in response to an initial stimulus is also rapidly turned off so that balanced amounts of pro- and anti-inflammatory molecules are achieved [124]. This layer of regulation also operates to control the amount of IL-10 produced, being mostly studied in macrophages and dendritic cells, where both the IL-10 mRNA stability and the expression of specific microRNAs (miRs) are known to be regulated [9]. As for IL-10-producing CNS cells (Fig. 2), previous reports demonstrate that the production of IL-10 upon TLR4 stimulation is regulated by miRs. Indeed, the inhibition of miR-466l-3p upon LPS challenge of microglia is described to lead to an upregulation in the IL-10 production by these cells [125]. In astrocytes, the overexpression of another miR, miR-181, was found to enhance the amount of LPS-induced IL-10 production [126].

Importantly, several mechanisms operate as negative feedback loops to restrain IL-10 production by CNS cells (Fig. 2). Activation of the signalling cascade mediated by glycogen synthase kinase (GSK)-3 functions as an endogenous mechanism to inhibit IL-10 production, whilst enhancing the production of pro-inflammatory cytokines, by microglial cells upon TLR4 activation [127]. In line with this, abrogation of GSK-3, through chemical inhibitors or siRNA, was shown to restore TLR4-induced IL-10 production in microglia with a concomitant reduction in the levels of pro-inflammatory mediators [82, 128]. Furthermore, blockade of GSK-3 was shown to induce p38 and ERK, thus confirming the role for these MAPKs in enhancing IL-10 production [82]. A similar role for GSK-3 in regulating IL-10 was previously demonstrated for other immune cell types [6, 9]. In both microglia and astrocytes, IL-10 was found to down-regulate its own transcription and that of the IL-10R when exogenously provided to untreated and LPS-treated cells [41].

## Conclusions

The importance of the innate immune cells of the CNS to maintain the brain homeostasis is now fully accepted. In this context, the instrumental role of microglia for brain development and functionality is unquestionable [21]. Whether microglia activation is also instrumental for pathogen elimination, or whether mononuclear cells from the periphery do this job, remains unclear. In any case, the immune response triggered in the brain is critical to restore homeostasis upon injury. However, above a certain threshold, the initially immune-protective response may become immune-degenerative, by causing tissue damage. Given the demonstrated potential of IL-10 in modulating brain inflammatory settings, it is of major importance to understand how IL-10 production is regulated in innate immune cells of the CNS and how it impacts inflammatory responses in this compartment. Thus, unveiling the common and the cell-specific mechanisms regulating IL-10 production in different settings and by different cellular populations will open new avenues for the development of specific targets to effectively and efficiently modulate IL-10. For this, the development of more suitable animal models, for example cell-specific genetic manipulation of IL-10, as well as deeper molecular studies of the processes underlying IL-10 expression and secretion, are required. Also of utmost importance is the understanding of the temporal and spatial dynamics of IL-10 production and action, so that the design of immune interventions may be optimized. This knowledge will potentiate the use of immunomodulatory, anti-inflammatory therapies targeting IL-10 production in several neurodegenerative conditions where inflammation is harmful.

## Abbreviations

APP: Amyloid precursor protein
CNS: Central nervous system
DAMP: Damage-associated molecular pattern
ERK: Extracellular signal-regulated kinase
GSK: Glycogen synthase kinase
IFN: Interferon
IL: Interleukin
JAK: Janus kinase
JNK: c-Jun N-terminal kinase
LPS: Lipopolysaccharide
MAPK: Mitogen-activated protein kinase
MEF2D: Myocyte enhancer factor 2D
MHC-II: Major histocompatibility complex class II
miR: MicroRNAs
NF: Nuclear factor
PAMP: Pathogen-associated molecular pattern
PI3K: Phosphatidylinositol-4,5-bisphosphate-3-kinase
PKR: Protein kinase R
PRR: Pattern recognition receptor
STAT: Signal transducer and activator of transcription
TGF: Transforming growth factor
Th: T helper
TLR: Toll-like receptor
TNF: Tumour necrosis factor
